# An analysis of the factors affecting children and adolescent lifestyle in South Korea: A cross-sectional study with KCYPS 2018

**DOI:** 10.1371/journal.pone.0263923

**Published:** 2022-02-17

**Authors:** Ah-Ram Kim, Seohyun Lee, Ji-Hyuk Park

**Affiliations:** 1 Department of Occupational Therapy, Graduate School, Yonsei University, Wonju, South Korea; 2 Department of Global Public Administration, College of Humanities and Social Sciences Convergence, Yonsei University, Wonju, South Korea; 3 Department of Occupational Therapy, College of Software and Digital Healthcare Convergence, Yonsei University, Wonju, South Korea; 4 Super-Aged Society New Normal Lifestyle Research Institute, Yonsei University, Wonju, South Korea; Gazi University, Faculty of Health Sciences, TURKEY

## Abstract

**Objective:**

The purpose of this study is to identify lifestyle factors that affect the subjective health conditions of adolescents in South Korea by utilizing data from the Korean Children’s and Youth Panel Survey 2018 conducted in Korea.

**Methods:**

This cross-sectional study used data from 4,490 students (2,399 students in the fourth grade of elementary school and 2,541 students in the first grade of middle school). The data obtained were evaluated using living time (sleep time, study time, leisure time), emotions (happiness, self-esteem, emotional problems), capabilities (cooperative attitude, grit), health (life satisfaction, exercise time, number of breakfasts, quality of sleep), media (smartphone use and dependence), school (school life satisfaction, relationship with friends and teachers), and home (time spent with parents, parenting attitude).

**Results:**

We confirmed that regardless of grade, living time (leisure time), emotion (happiness, self-esteem, emotional problems), capabilities (grit), health (life satisfaction, exercise time, quality of sleep), media (smartphone dependence), and school (school life satisfaction, friendly, and teacher relations) are related to subjective health conditions. These results confirmed that evaluation parameters are related to subjective health conditions regardless of grade.

**Conclusion:**

This investigation revealed that the higher the quality of sleep enhanced positive emotions while increased exercise

## Introduction

Adolescence is a key transitional stage in which lifestyle is formed, which determines the health, well-being, and quality of adult life [1]. However, it is also a time for frequent unhealthy behaviors [2]. such as lack of physical activity, excessive use of screen time, skipping breakfast, and lack of sleep [2, 3]. Some adolescent lifestyle changes are temporary, while in some cases the adolescent lifestyle tends to last until adulthood [4]. The study of health-related quality of life (HRQoL), especially, was initially conducted in adults. However, as a factor affecting the overall life of adolescence, early intervention has been highlighted as an important concern [5]. In the absence of serious health threats or life events, HRQoL is generally considered stable throughout adolescence [6, 7], although some studies have shown a decline in HRQoL during adolescence [8–10]. Given the risks of psychological, mental, and physical health problems appearing in adolescence leading to adulthood, identifying and solving the early signs of HRQoL reduction can provide adolescents with opportunities to stay healthier in adolescence [11].

The World Health Organization (WHO) defines lifestyle as "a specific type of behavior that can reduce disease and early death by personal physical, mental, and social interaction."[12]. Lifestyle is a concept that includes the overall aspects of life, such as individual ways of thinking, living, and activities, and is specifically subdivided into items such as eating habits, daily life, and physical activity [13]. It is also closely related to health and healthy practices such as physical activity and maintaining a healthy diet. Lifestyle helps in improving the quality of life; it is not a treatment but a means of disease prevention and management [14].

These preliminary studies support the notion that adolescents are vulnerable to unhealthy lifestyles [15, 16]. Several major health-related behaviors, such as alcohol consumption, insufficient physical activity, long-term sitting behavior, obesity, poor diet, and tobacco abuse, often appear or develop during adolescence and increase the risk of non-communicable diseases (NCD) in the future [17]. Thus, a comprehensive understanding of the lifestyle factors of adolescence is essential to improve the health of adolescents and to reduce the risk of follow-up diseases in adulthood [18].

According to prior studies, these factors are widespread among adolescents [15, 16]. In addition, exposure to various risk factors increases the likelihood of poor subjective health and poor quality of life [19]. Subjective health is individual’s subjective perception of own health status and constitutes as essential predictive indicator for later morbidity, mortality, school-dropout, use of social welfare [20]. Therefore, it is important to understand the lifestyle factors of children and adolescents in Korea to ensure their well-being. Due to the lack of adequate research on adolescent lifestyle issues within Korea, this study has attempted to apply international research findings on children and adolescents to assess the factors affecting the lifestyle of Korean youth. Therefore, the purpose of this study was to identify the factors influencing the lifestyle of Korean children and adolescents.

## Methods

The data used in this study were obtained from the Korean Children and Youth Panel Survey 2018 (KCYPS 2018) conducted by the National Youth Policy Institute. KCYPS 2018 aimed to establish panel data that could comprehensively identify changes in the growth and development of children and teenagers, with a view to provide basic information for child- and youth-related policies and academic research [21]. It also offered a holistic perspective on the various factors related to Korean youth [22]. A total of 5,197 students (fourth grade elementary school (E4) and first grade middle school (M1) from 333 schools participated in the survey. In this study, missing values were excluded, and finally, 2,399 elementary school students and 2,541 middle school students were participants. This study was conducted after obtaining approval from Yonsei University’s Institutional Review Board in accordance with research ethics (Approval Number: 1041849-202101-SB-005-01).

### Living time

Living time refers to time earmarked for sleep, study, and leisure. For this factor, certain questions from the 2013 Youth Media Usage were revised and supplemented [21]. To assess sleep time factors such as bedtime and wake-up time were studied. Time allotted for study was inclusive of after-school classes, private tutoring, online and/or TV lectures, self-study, and reading sessions. Leisure time constituted hours spent for exercise and physical activity, time spent for smartphone and computer usage, watching TV, and hours spent for playing with friends.

### Emotion

Under the category of emotions, happiness, self-esteem, and emotional problem were the variables used for the study. Measurement of happiness was based on relevant questions extracted from the Korea Institute of Child Care and Education. The higher the score, the higher the feeling of happiness is interpreted [23]. The sense of self-esteem was understood in terms of perception by society and self-image. The higher the score, the higher the feeling of self-esteem is interpreted [24]. The Korean Symptom Check List (KSCL 95) was used to frame questions related to emotional problem. KSCL 95 evaluates attention, aggression, physical symptoms, social atrophy, and depression, and it is interpreted that the higher the score, the more serious the emotional problem [25].

### Capabilities

The capabilities of competency included cooperative attitude and grit. The capabilities of competency included cooperative attitude and grit. Measurement of cooperative attitude was based on relevant questions extracted from the International Association for the Evaluation of Educational Achievement (IEA) International Civic and Citizenship 2009 (ICCS) Asia Regional Module (ARM) [26]. Measurement of grit was based on the Korean Grit Scale for Children [27]. The higher the total score, the high the cooperative attitude and grit.

### Health

Youth health was studied based on subjective life satisfaction, exercise time, number of breakfasts, and BMI [24]. The tool of life satisfaction measurement used was the Satisfaction with Life Scale (SWLS). The higher the total score, the higher life satisfaction [28]. Exercise time have been investigated for sweaty hours during the past week. Days on which breakfasts were had were taken into consideration to assess the frequency of consuming the meal. Height, weight, and other related statistics were collected to calculate BMI. As for the quality of sleep, the subjective quality of sleep felt by the participant was measured, and a four-point Likert scale was used.

### Media

Sub-items of media included smartphone use and smartphone dependence. As for whether or not to use a smartphone, it was measured whether to use a smartphone is currently used or not. The dependence on a smartphone was examined through a set of 15 questions on self-diagnosis scale pertaining to gadget addiction, It is interpreted that the higher the score, the higher the dependence on smartphones [29].

### School

As for school life satisfaction, satisfaction with school life was investigated last semester, and it was answered on a 5-point Likert scale (1 = very dissatisfied; 2 = dissatisfied; 3 = normal; 4 = satisfied; 5 = very satisfied). Friendly relationships were measured through the Peer Relationship Quality Scale for Adolescents [30]. Teacher relationships were measured through the Student-Teacher Attachment Relationship Scale (STARS) [30]. The higher the score, the more positive the relationship with friends and teacher is interpreted.

### Home

The time spent with parents was measured for conversation time with parents (or caregivers). Parenting attitudes were measured using the Parents as Social Context Questionnaire for Adolescents (PSCQ_A) [31].

### Data analysis

The study was conducted based on data from the 2018 Korean Children and Youth Panel Survey. Data pertaining to 208 participants from fourth grade elementary school and 49 from first grade middle school panel were missing and these have been excluded from the analyses without any major impact on the results. Demographic information was frequently analyzed using technical statistics to examine the general characteristics of the study participants. We compared demographic differences between boys and girls, using an independent t-test. Correlation analysis was performed to determine the simple correlation between variables and multicollinearity between the independent variables. Correlation factors were analyzed using stepwise regression to examine the association of predictive factors related to subjective health conditions manipulated by nominal variables. IBM SPSS (version 22.0; IBM Corp., Armonk, NY, USA) was used for all analyses.

## Results

### Demographic characteristics

The KCYPS 2018 sample comprised 2,607 students in the fourth grade of elementary school and 2,590 students in the first grade of middle school. Excluding missing data, 2,399 elementary school seniors and 2,541 middle school freshmen were analyzed (Table 1). In the fourth grade of elementary school, there were 1,193 boys and 1,206 girls, while in the first year of middle school, there were 1,375 boys and 1,166 girls. The respondents were primarily urban residents. The average sleeping time was 552 minutes for boys and 548 minutes for girls from elementary school, and 489 minutes for boys and 464 minutes for girls from the middle school. Both elementary and middle school students answered positively to the query on "Tend to sleep well" and there was no noticeable gender disparity. In terms of subjective health, male students in elementary school said they were "very healthy" (47.5%) and female students answered "healthy side" (50.1%). In middle school, the majority of the respondents said they were "healthy" (boys 51.3% and girls 58.6%), showing slight gender difference. With regard to exercise time, most boys in elementary and middle schools responded "4 hours over" (50.9% for elementary and 34.9% for middle school), while majority of girls from elementary school chose "1 hour" (29.7%) and those from middle school mostly responded to "None" (30.4%). The number of breakfasts averaged 5.8 for boys in elementary school and 5.9 for girls, showing no gender difference. Middle school boys averaged 5.1 times and female students 4.7 times, showing gender differences. With regard to BMI, both elementary and middle school respondents had the highest normal weight.

**Table 1:** Demographic characteristics (N = 4,940).

			E4 (n = 2,399)			M1 (n = 2,541)		
			Boy (n = 1,193)	Girl (n = 1,206)	p	Boy (n = 1,375)	Girl (n = 1,166)	p
Residential area	Metropolis		501(42.0)	499(41.4)	0.953	637(46.3)	507(43.5)	0.290
	Small and medium city		494(41.4)	504(41.8)		552(40.1)	483(41.4)	
	Town and village		198(16.6)	203(16.8)		186(13.5)	176(15.1)	
Living time	Sleep time (minute)		552±49	548±48	0.470	489±54	464±59	0.001
	Study time (average*)		3.01	3.16	0.016	2.95	2.95	0.829
	Leisure time (average*)		2.95	2.81	0.003	3.15	2.92	0.001
Health	Exercise time	None	58(4.9)	151(12.5)	0.001	116(8.4)	354(30.4)	0.001
		1 hour	180(15.1)	358(29.7)		273(19.9)	324(27.8)	
		2 hours	176(14.8)	215(17.8)		257(18.7)	211(18.1)	
		3 hours	172(14.4)	186(15.4)		249(18.1)	146(12.5)	
		4 hours over	607(50.9)	296(24.5)		480(34.9)	131(11.2)	
	Number of breakfasts		5.8	5.9	0.896	5.1	4.7	0.008
	Quality of sleep	Can’t sleep very well	17(1.4)	9(0.7)	0.281	11(0.8)	17(1.5)	0.061
		Can’t sleep	110(9.2)	101(8.4)		154(11.2)	159(13.6)	
		Tend to sleep well	627(52.6)	663(55.0)		784(57.0)	663(56.9)	
		Sleep very well	439(36.8)	433(35.9)		426(31.0)	327(28.0)	
	BMI	Under weight	59(4.9)	77(6.4)	0.001	63(4.6)	51(4.4)	0.001
		Normal	851(71.3)	919(76.2)		1,029(74.8)	954(81.8)	
		Over weight	77(6.5)	121(10.0)		86(6.3)	95(8.1)	
		Obesity	206(17.3)	89(7.4)		197(14.3)	66(5.7)	
Subjective health conditions	Not healthy		9(0.8)	2(0.2)	0.088	10(0.7)	3(0.3)	0.001
	Not very healthy		57(4.8)	53(4.4)		100(7.3)	93(8.0)	
	Healthy side		560(46.9)	604(50.1)		705(51.3)	683(58.6)	
	Very healthy		567(47.5)	547(45.4)		560(40.7)	387(33.2)	

*1 = not at all, 2 = less than 30 minutes, 3 = 30 minutes to less than an hour, 4 = less than 1 to 2 hours

### The correlation between subjective health conditions and factors

In the case of elementary school students, their living time (leisure time), emotions (happiness, self-esteem, emotional problems), capabilities (grit), health (life satisfaction, exercise time, quality of sleep), media (smartphone dependence), school (school life satisfaction, friendly relationship, teacher relationship), and home (parenting attitude) were highly correlated with subjective health conditions (p < .001) (Table 2). Living time (study time) and health (number of breakfasts, BMI) were significantly correlated with subjective health conditions (p < .05), living time (sleep time), capabilities (cooperative attitude), media (smartphone use), and home (time spent with parents) showed no significant correlation (p>.05). These results show that the higher the study time and leisure time among living time, the higher the subjective health condition. In addition, it can be seen that the higher the happiness, self-esteem, and grit, the less emotional problems, the higher the subjective health condition. In terms of health, life satisfaction, exercise time, number of breakfasts were higher and quality of sleep, and the lower the BMI, the higher the subjective health condition. It was found that the lower the smartphone dependence and the better the school (satisfaction of school life, friendly relationship, teacher relationship) life, the higher the subjective health status. In terms of home, the more positive the parenting attitude of parents, the higher the subjective health condition.

**Table 2:** The correlation between subjective health conditions and factors.

		Subjective health conditions			
		E4		M1	
		t	p-value	t	p-value
Living time	Sleep time	0.035	0.086	0.026	0.182
	Study time	0.040*	0.049	0.060**	0.003
	Leisure time	0.059**	0.004	0.059**	0.003
Emotion	Happiness	0.284**	0.001	0.330**	0.001
	Self-esteem	0.347**	0.001	0.358**	0.001
	Emotional problem	-0.330**	0.001	-0.323**	0.001
Capabilities	Cooperative attitude	0.259	0.001	0.228**	0.001
	Grit	0.272**	0.001	0.276**	0.001
Health	Life satisfaction	0.300**	0.001	0.312**	0.001
	Exercise time	0.198**	0.001	0.197**	0.001
	Number of breakfasts	0.048*	0.020	0.086**	0.001
	BMI	-0.045*	0.026	-0.044*	0.027
	Quality of sleep	0.207**	0.001	0.193**	0.001
Media	Smartphone use	0.021	0.314	0.033	0.101
	Smartphone dependence	-0.203**	0.001	-0.182**	0.001
School	Satisfaction of school life	0.185**	0.001	0.232**	0.001
	Friendly relationship	0.251**	0.001	0.293**	0.001
	Teacher relationship	0.222**	0.001	0.221**	0.001
Home	Time spent with parents	0.036	0.075	0.073**	0.001
	Parenting attitude	0.282**	0.001	0.040	0.768

*p<0.05

**p<0.01

In the case of middle school students, living time, emotion, capabilities, health, media, school, and family were highly correlated with subjective health conditions (p < .005). Health (BMI) showed a significant correlation with subjective health conditions (p < .05). Living time (sleep time), media (smartphone use), and home (parenting attitude) were not significantly correlated (p>.05). These results show that the higher the study time and leisure time among living time, the higher the subjective health condition. In addition, it can be seen that the higher the happiness, and self-esteem, the less emotional problems, the higher the subjective health condition. In terms of capabilities, the higher the cooperative attitude and grit, the higher the subjective health status. In terms of health, life satisfaction, exercise time, number of breakfasts were higher and quality of sleep, and the lower the BMI, the higher the subjective health condition. It was found that the lower the smartphone dependence and the better the school (satisfaction of school life, friendly relationship, teacher relationship) life, the higher the subjective health status. In terms of home, the higher the time spent with parents, the higher the subjective health condition.

### Factors affecting subjective health conditions of fourth graders in elementary school

Stepwise regression analysis was performed to determine the effects on subjective health conditions. Prior to the regression analysis, we performed a correlation analysis to identify the factors associated with subjective health conditions (Table 3). This study found that living time, emotion, health, media, and school had a significant impact on subjective health conditions (p < .001). The explanatory power (R^2^) of the independent variable for subjective health conditions was 18.7%. The -2 log-likelihood and chi-square (x^2^) values for confirming the model’s fit were significant at 35.413 and 166.417, respectively (p < .001), which indicated that the model was suitable.

**Table 3:** Factors affecting subjective health conditions of fourth graders in elementary school.

		T	p-value
Living time	Study time	-0.531	0.595
	Leisure time	2.743*	0.006
Emotion	Happiness	4.839***	0.001
	Self-esteem	5.193***	0.001
	Emotional problem	-4.906***	0.001
Capabilities	Grit	0.313	0.754
Health	Life satisfaction	1.833	0.067
	Exercise time	6.370***	0.001
	Number of breakfasts	-1.055	0.291
	BMI	-1.389	0.165
	Quality of sleep	4.980***	0.001
Media	Smartphone dependence	-2.444*	0.015
School	Satisfaction of school life	1.295	0.195
	Friendly relationship	1.511	0.131
	Teacher relationship	2.812**	0.005
Home	Parenting attitude	1.571	0.116
AdjustR^2^		18.7	
-2 log likelihood		35.413	
model x^2^		166.417**	

*p<0.05

**p<0.01

***p<0.001

### Factors affecting subjective health conditions of first-year middle school students

Stepwise regression analysis was performed to determine the effects on the subjective health conditions. Prior to the regression analysis, we performed a correlation analysis to identify the factors associated with subjective health conditions (Table 4). This study found that living time, emotion, health, media, and schools (had significant effects on subjective health conditions (p < .001). The explanatory power (R^2^) of the independent variable for subjective health conditions was 19.8%. The -2 log-likelihood and chi-square (x^2^) values for confirming the model’s fit were significant at 37.732 and 199.343, respectively (p < .001), which indicated that the model was suitable.

**Table 4:** Factors affecting subjective health conditions of first-year middle school students.

		T	p-value
Living time	Study time	-0.213	0.832
	Leisure time	0.633	0.527
Emotion	Happiness	6.839***	0.001
	Self-esteem	4.538***	0.001
	Emotional problem	-3.764***	0.001
Capabilities	Cooperative attitude	-0.215	0.830
	Grit	1.847	0.065
Health	Life satisfaction	0.720	0.472
	Exercise time	6.422***	0.001
	Number of breakfasts	0.208	0.836
	BMI	-2.342	0.019
	Quality of sleep	3.522	0.001
Media	Smartphone dependence	0.290	0.772
School	Satisfaction of school life	2.284*	0.022
	Friendly relationship	5.331***	0.001
	Teacher relationship	0.918	0.359
Home	Time spent with parents	0.640	0.522
AdjustR^2^		19.8	
-2 log likelihood		37.732	
model x^2^		199.343**	

*p<0.05

**p<0.01

***p<0.001

## Discussion and conclusion

The purpose of this study has been to analyze the factors that influenced the lifestyle and subjective health condition of children and adolescents in Korea with reference to studies on adolescent lifestyle conducted outside the country. To this end, technical statistical analysis was conducted on subjective health conditions and lifestyle factors using the KCYPS 2018 data, and regression analysis was done to determine the influence of factors identified in the correlation between factors affecting subjective health conditions.

Some of the common factors that affect the subjective health status of both the groups (E4, M1) are quality of sleep, emotional factors, and exercise. Regarding school life satisfaction, there are differences between the two groups. While the elementary school group (E4) was more affected by relationship with the teacher, the middle school group (M1) gave more emphasis to relations with friends. With regards to health, it was found that better subjective health condition corresponded to higher level of satisfaction with life. According to a previous study targeting elementary school students, the relationship with the teacher was found to have a significant positive correlation with subjective health [32]. This is consistent with the research results that the emotional support of teachers is closely related to the subjective health of children [33]. On the other hand, adolescence is important for adolescents as the number of interpersonal relationships increases compared to childhood, spending a lot of time with peers, and peer relationships affect not only school but also personal growth and development [34, 35]. According to a previous study, the more positive the students perceived their relationship with their peers, the higher their subjective well-being [36]. These findings suggest that factors influencing subjective health may change as teens grow. However, whether these differences are limited to the group participating in this study or reflect actual differences needs to be confirmed through continuous research.

According to the results of this study, the quality of sleep is directly proportional to subjective health. Sleep is an essential and universal function in humans [37]. Poor sleep quality has a negative effect on other areas related to physical health such as chronic pain [38] and higher body mass index [39, 40], among other negative outcomes. Poor sleep quality is also associated with negative psychological effects such as anxiety and depression [41], aggression [42], and attention deficit/overactivity disorder [43]. As such, it can be seen that sleep quality is an important factor for subjective health and suggests the need for further research related to sleep quality in adolescents.

With regard to emotions, it was found that the better the positive emotions such as happiness, self-esteem, and sense of cooperation, the more subjective health conditions was proportionally increased. Adolescence is a vulnerable period in which developing and maintaining healthy social and emotional habits are important for mental health and well-being. This is a crucial period when tremendous physical and mental changes happen which place adolescents at high risk for mental health conditions [44]. Prior studies have found a link between poor mental health in childhood and adolescence, poor quality of life and loneliness [45], higher criminal behavior [46], and worse mental and physical health in adulthood [45, 47]. As it is an important time for the formation of emotional habits, it is believed that an emotional mediation approach is necessary to improve the subjective health of adolescents.

This study has further shown that increased time spent in exercising also enhances the subjective health condition. Prior research has found that greater involvement in physical activities and less leisure-time screen use contribute to fewer depressive symptoms in adolescents [48]. Physical exercise is especially important for adolescents’ health because it can help them disperse from infectious disease concerns, reduce cortisol secretion, and increase endorphin secretion [49]. To improve the mental and physical health of teenagers, it is necessary to improve the curriculum at the national level to incorporate more time for exercise or physical activities.

According to the study, relationships with teachers have an impact on the subjective health condition of elementary school students while relationships with friends affect middle school students’ health conditions. According to a previous study targeting elementary school students, the relationship with the teacher was found to have a significant positive correlation with subjective health [32]. In the case of adolescents, since many physical activities are performed in the context of social peer groups among adolescents, relationships with friends can be considered a major variable for health [50]. Moreover, support from friends has been shown to be more important than parental support in this age group [51]. Prior studies in the area of physical activity and health have also reported the importance of friends as supporting factors for adolescents [52, 53]. However, the study confirms that the factors that affect elementary and middle school students are different. Earlier studies in this area were mostly aimed at teenagers whereas the present study has taken into consideration different age groups and so, there have been differences in the results of the study. To better understand and identify factors related to youth’s health and school life, it is believed that more research, across age groups, will be needed to identify the impact factors.

Health-related lifestyles consist of patterns of interaction of health-related behaviors, directions, and resources coordinated by individual groups on social, cultural, and economic environments [54]. Adolescence repeats various types of unhealthy behaviors, which could potentially jeopardize the health of adolescents, requiring the study of health-related lifestyles [55]. Such research requires a multifaceted and objective evaluation tool that focuses on youth lifestyle. However, there are no standardized evaluation tools for teenagers in South Korea. It is necessary to develop standardized evaluation tools to provide interventions and related health services to build a healthy lifestyle for adolescents.

This study had some limitations. First, this study used data that was measured using a self-reporting questionnaire. It could measure the attitude that teenagers feel, but not their attitude to upbringing. In addition, there was a possibility that teenagers might reduce or deny their own problems for negative indicators such as smartphones. To overcome these limitations in the future, concurrent survey and evaluation of parents and children would provide high validity. Second, this study was analyzed with data from a specific point in time using a cross-sectional study. This provided insight into the current situation at that point, but not the change over time. In the future, a follow-up study is needed to determine whether the factors change over time. Third, the subjects of the data used in this study were elementary and middle school students. They could not be regarded as representative of Korean youth as all age groups were not considered. In the future, research should be conducted on teenagers of various ages.

Nevertheless, we analyzed nationally representative data, which could enhance the generalizability of our findings. this study is meaningful in that it examined the lifestyle factors that affect the subjective health of Korean teenagers, and based on this, it offers an understanding of current events and discussions on youth lifestyle. The findings of this study is important for public health decision makers and healthcare practitioners to plan and develop appropriate intervention and prevention programs for a healthy lifestyle among adolescents. In future studies, it is necessary to confirm the lifestyle predictors of adolescents through more accurate measurement of the factors identified in this study.
